# Spotlight on early-career researchers: an interview with César de la Fuente

**DOI:** 10.1038/s42003-020-1003-4

**Published:** 2020-06-05

## Abstract

Dr. César de la Fuente is a Presidential Assistant Professor at the University of Pennsylvania. He leads a Machine Biology group developing computational tools to expand the antibiotic arsenal, engineer the microbiome and study and control brain function and behavior. His work has been recognized by the Langer Prize, ACS Kavli Emerging Leader in Chemistry award, ACS Infectious Diseases Young Investigator Award, STAT News, GEN, and the MIT Technology Review. We asked Dr. de la Fuente about his research and journey of the field as part of our series on early-career researchers.

**Figure:**
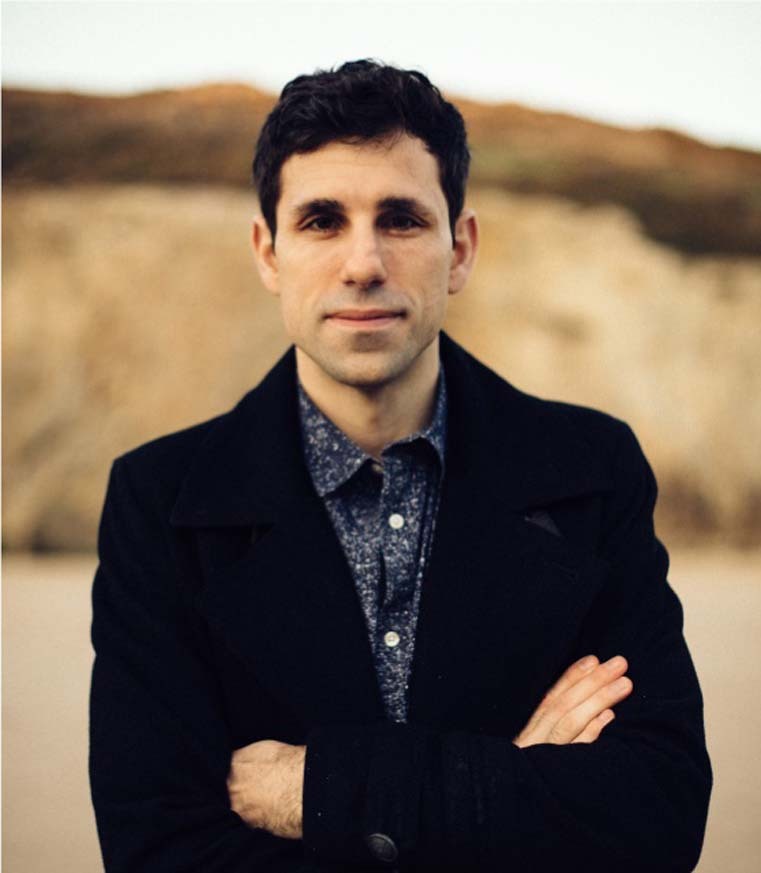
Diana Fontenla

Please tell us about your research interests.

I lead the Machine Biology Group at the University of Pennsylvania (UPenn). Our goal is to develop antibiotics by means of computers. In order to achieve this, we leverage a highly interdisciplinary approach involving principles and concepts from artificial intelligence (AI), synthetic biology, chemistry and microbiology. This work will be important as it is estimated that 10 million people will die annually as a result of antibiotic-resistant infections (i.e., 1 death every 3 s).

What are some ways biologists can learn from engineers, and vice-versa?

The boundary among disciplines is fading. In the Machine Biology Group, we believe in ‘transdisciplinarity’ which entails the convergence of a wide range of disciplines, from chemistry and physics to synthetic biology, microbiology and computer science. Biologists tend to have a holistic view of the world and aim to understand the biological systems, whereas engineers see the world from a very practical perspective and aim to build new and useful tools. I am fascinated by the intersection of both fields as we can use the knowledge learned from complex systems in biology to control it and build a new system. I believe that innovations at this intersection will allow us to tackle big problems.

What has your journey been up to this point?

Ever since I was a child, I have been fascinated by the world around me. This passion has been a constant throughout my career and drove me to pursue my PhD degree (UBC, 2014). My goal was to (1) understand, (2) control, and (3) computerize biological systems. I focused on the simplest organisms (bacteria) and the workhorses of life (proteins). I learned how bacteria become harmful and built in the lab tiny proteins called antimicrobial peptides to target bacteria.

I loved the chemistry of proteins for the intuition it yielded about the nature of molecules to enable life. Proteins are the workhorses of life and have unparalleled complexity in terms of their sequence variability. I reasoned that they were the perfect raw material for creating useful tools for deciphering diseases, such as those caused by bacteria. I decided that we had collected sufficient information to take the leap from understanding antimicrobial peptides to trying to control them. The next step would involve optimized control of peptide design, and subsequently teaching computers this information, to enable molecular discovery to solve all kinds of problems.

Molecular biology felt too much like a recipe book leaving little space for creativity. On the other hand, by controlling biology we could open the door for machines and automation to achieve molecular discovery and functional screening. Humans would be left to do the thinking.

I then moved to MIT in 2015, at a time when we were starting to realize the potential for computers to revolutionize biology by their ability to process huge masses and fluxes of data. Its application in antibiotic discovery was still in the infancy stage. Soon thereafter, we made progress in understanding multi-level relationships of antimicrobial peptides, engineered living cells to generate peptides at a low cost and high efficiency, and developed algorithms capable of discovering antibiotics in proteomes. Next, my colleagues and I focused on developing the first computer-made antibiotic. We arrived at the core idea of digitizing peptides and evolving them using evolutionary theory by going through the tree of life and thinking about which molecules had antimicrobial qualities, as well as the processes needed to encode them using machines.

Ultimately, I was recruited by the University of Pennsylvania as a Presidential Professor where I work with a team to develop computer-made tools and medicines. By merging synthetic biology, chemistry, physics, and computer science, we can now devise strategies against antibiotic resistance that nature has not yet discovered.

Can you speak of any challenges that you have overcome? What advice would you give to your younger self?

I have had to overcome a number of challenges over the years. I was never a great student and never did well with memorization-based learning; however, I was always very curious about the world around me and wanted to help others. I believe that pursuing this curiosity helped me overcome my limitations as a student in our current education system. I would encourage my younger self to feel comfortable and embrace the feeling of vertigo that comes with entering the unknown. It is all part of the learning process. Indeed, this feeling is in fact very common in science, especially when we step into new fields.

How do you value mentoring in your career?

Mentoring others is a crucial component of my daily life. I am truly committed to encouraging the inclusion of historically underrepresented groups in the STEM fields. I put specific emphasis on achieving gender parity in my lab and on recruiting exceptional individuals that come from different and often unconventional backgrounds. There is much value in recruiting people that are incredibly talented, creative, driven and passionate and who perhaps were not given previous opportunities. Nearly all current members of my lab are from underrepresented minorities. I encourage others to do the same so we can shift the current academic landscape and promote diversity and inclusion. My goal is to mentor and empower the next generation of women and underrepresented minorities, so their portraits decorate the hallways of universities and institutions in the years to come.

What is something people may not know about synthetic biology?

Synthetic biology is advancing at an accelerated rate with the help of computation and AI. I believe that the future of synthetic biology lies at this intersection.

*This interview was conducted by Associate Editor Faten Taki and Chief Editor Brooke LaFlamme*.

